# Investigation into the Effects of Allergen Exposure and Topical Vinegar and Water Spray on Skin Barrier Parameters in Atopic Dogs

**DOI:** 10.3390/vetsci11100459

**Published:** 2024-10-01

**Authors:** Rosanna Marsella

**Affiliations:** College of Veterinary Medicine, University of Florida, Gainesville, FL 32610, USA; marsella@ufl.edu

**Keywords:** pH, TEWL, canine atopic dermatitis

## Abstract

**Simple Summary:**

Skin barrier dysfunction plays an important role in allergic skin diseases. This study aimed to monitor changes in skin parameters such as pH and water loss through the skin during the course of an allergy flare in a colony of allergic dogs. The dogs were known to be allergic to dust mites and they were exposed to dust mites twice weekly for two weeks to induce and allergy flare while monitoring skin parameters daily. We found that skin pH and water loss through the skin both increased during the allergy flare and that they were correlated to the severity of the dermatitis. We then wanted to see if the daily application of a vinegar water spray could help lower pH and improve dermatitis despite the exposure to the dust mites. We found that vinegar water treatment once daily was not sufficient to maintain a lower pH and decrease the severity of dermatitis when the dogs were exposed to dust mites. We conclude that skin pH is important in allergic dermatitis but that strategies other than vinegar and water should be tried to lower skin pH.

**Abstract:**

Increased skin pH and transepidermal water loss (TEWL) are documented in atopic people and dogs but no study has investigated how these parameters change during an allergy flare. Our primary aim was to challenge atopic beagles to dust mites and measure pH and TEWL during a flare of atopic dermatitis and correlate these parameters to clinical signs. A secondary aim was to evaluate in a randomized placebo-controlled study whether the daily application of 50/50 vinegar spray improves clinical signs and affects skin parameters despite the allergen challenge. Fifteen atopic dogs were challenged epicutaneously twice weekly for 2 weeks with allergen application on the inguinal and medial thigh areas. The severity of dermatitis was scored daily (CADESI-03). TEWL and pH were measured daily on the inguinal and medial thigh areas. A repeated measures ANOVA showed the significant effect of time, with increased pH (*p* < 0.0001), TEWL (*p* < 0.0001), and CADESI (*p* < 0.0001) during allergen challenge. Significant positive correlations were found between CADESI and pH (r = 0.3556; *p* < 0.0001), CADESI and TEWL (r = 0.36; *p* < 0.0001), and pH and TEWL (r = 0.45; *p* < 0.0001). Daily application of 50/50 vinegar did not improve dermatitis, pH, and TEWL compared to the control treatment. It can be concluded that both pH and TEWL are markers of disease severity in canine atopic dermatitis.

## 1. Introduction

The critical importance of skin barrier in atopic dermatitis has been widely documented in recent years [1,2]. Whether skin barrier impairment is primary and linked to genetic mutations or is due to increased exposure to pollutants [3,4], there is increasing evidence that a disrupted skin plays a role in both sensitization and in the perpetuation of allergic disease through vicious progressive cycles of inflammation and barrier disruption. For these reasons, it is critical to learn more about how skin barrier function changes in the course of an allergy flare and what can be done to minimize skin barrier disruption.

Indicators of a disrupted skin barrier are increased pH and increased transepidermal water loss (TEWL) [5,6]. The acidity of the skin is of critical importance for homeostasis and proper barrier function [7,8]. An acidic pH is vital to control proper desquamation and to ensure the appropriate extrusion of the content of lamellar bodies to provide antimicrobial peptides and the precursors for lipid lamellae formation [9]. The activity of ceramidases and proteases is tightly controlled by pH and any sustained increase in pH leads to decreased synthesis of ceramides and accelerated desquamation, which physically disrupt skin barrier. Additionally, an acidic pH is necessary to minimize the overgrowth of pathogens and ensure the biodiversity of cutaneous microbiome with balanced populations of commensals [10]. The pH of human atopic skin is higher than healthy individuals in both lesional [11] and non-lesional skin [12]. This change seems to be peculiar to atopic dermatitis and not just secondary to any form of skin inflammation since psoriasis patients actually have a lower pH compared to healthy controls [13,14]. For this reason, the acidification of the skin is proposed as a treatment for atopic dermatitis [15,16]. Atopic dogs also show consistently higher pH values compared to normal dogs [17]. Skin pH seems to be the most repeatable measurement while the measurement of TEWL has been reported to have the highest intra- and interobserver variability [17]. Other investigators reported that the increased pH of atopic dogs’ skin correlates with the severity of dermatitis in some sites but not in others [18].

Increased TEWL has been documented in both canine and human atopic dermatitis [19,20]. However, the measurement of TEWL has shown large variability in the values from site-to-site and from day-to-day and in different individuals, even in non-allergic dogs [21]. Previously published results on the correlation between severity of dermatitis and TEWL have yielded mixed results [22], with correlations only at some sites [23]. Transepidermal water loss can be a tricky parameter to measure as many factors affect it [24], and it is affected by the skills of who is measuring it [25]; however, skin pH is quick and easy to measure [26]. The requirements for controlling ambient conditions in conjunction with the variability of TEWL diminish the practical clinical usefulness of this measurement.

Currently, there is no published study that assessed longitudinally how skin pH and TEWL change during the course of an allergy flare. Thus, the primary aim of our study was to investigate how pH and TEWL change in atopic dogs during the course of an allergy flare. For this purpose, we epicutaneously challenged atopic dogs with an allergen to which they were allergic and known to be clinically responsive [27]. We controlled their diets, the allergen challenge, and the environment, and we took multiple measurements to see specifically how pH and TEWL change while the dogs are challenged and the lesions of atopic dermatitis gradually develop. As acidification is advocated for atopic dermatitis, a secondary goal was to investigate whether the daily application of 50/50 vinegar water spray could improve clinical signs in atopic dogs undergoing allergen challenges. The efficacy was evaluated in a randomized, placebo-controlled trial by measuring the severity of clinical scores between treatment groups. The skin pH was measured during the allergen challenge and correlated with the severity of clinical signs.

## 2. Materials and Methods

All procedures in this study were approved by University of Florida IACUC (# 201810298).

### 2.1. Animals

Fifteen atopic beagles (six females and nine males; 5 years old) previously sensitized to house dust mites and known to flare upon re-exposure to the allergen were used.

### 2.2. Allergen Exposure

Dogs were challenged by epicutaneous exposure to Dermatophagoides farinae (25 mg/dog/day, twice weekly for two weeks (on days 2, 6, 10, and 13). House dust mites (HDM) were prepared from culture (natural Dermatophagoides farinae, Greer Laboratories Inc., Lenoir, NC, USA) and mixed with phosphate-buffered saline (PBS; pH 7.2) to a final concentration of 15.6 mg/mL, as previously reported [28]. Each dog received a total of 25 mg of HDM/challenge.

### 2.3. Scoring of Dermatitis

The severity of dermatitis was assessed daily using a validated scoring system (CADESI-03) [29] limited to the inguinal and medial thigh area. Daily pictures were taken from the inguinal area and medial thighs, right before TEWL, pH, and product spray application. Pictures were randomized and scored by the same investigator who was blinded regarding the timings of the pictures in relationship to the allergen challenge and the product allocation. On the days of challenge, dermatitis was scored twice, both before and 4 h after allergen exposure.

### 2.4. Assessment of Skin Barrier Parameters

Temperature and humidity settings were maintained at 25 ± 5 °C (mean ± SD = 24.7 ± 0.4 °C) and 50 ± 10% (median = 47%). Dogs were acclimated for 30 min prior to measurement.

TEWL was measured daily, on both the left and right of the dogs’ inguinal areas and medial thighs using a close chamber device (VapoMeter^®^, Delfin, Miami, FL, USA). Four measurements were taken per dog each day with three replicates each. The average was used for analysis.

Skin pH was measured every day on both sides of the inguinal area as well as the medial sides of both hind legs using the Skin-pH-Meter^®^ (Courage + Khazaka electronic GmbH, Köln, Germany). Four measurements per dog/day with ten replicates each. The last five values were used for calculations and the average was used for analysis.

#### 2.4.1. Treatments

Each dog received both the vinegar spray (50% white vinegar; 50% distilled water) on half of the inguinal/hind area and distilled water was used on the other side, for a total of 2 mL/dog/treatment. Sides were randomized between dogs so that each dog was its own control. Personnel carrying out the assessments were not aware of the randomization. Treatment was administered once daily after taking pictures and measuring TEWL and pH.

#### 2.4.2. Statistics

The Shapiro–Wilk test was used to check for normality. A repeated measures ANOVA was used to investigate the effect of allergen exposure on CADESI, TEWL, and pH. Dunnett’s multiple comparisons test was performed to compare each day to baseline. Correlations were assessed using Pearson’s correlation test. Significance was set at 0.05.

## 3. Results

The allergen challenge significantly increased pH, TEWL, and clinical scores.

### 3.1. pH and TEWL

**Figure 1:**
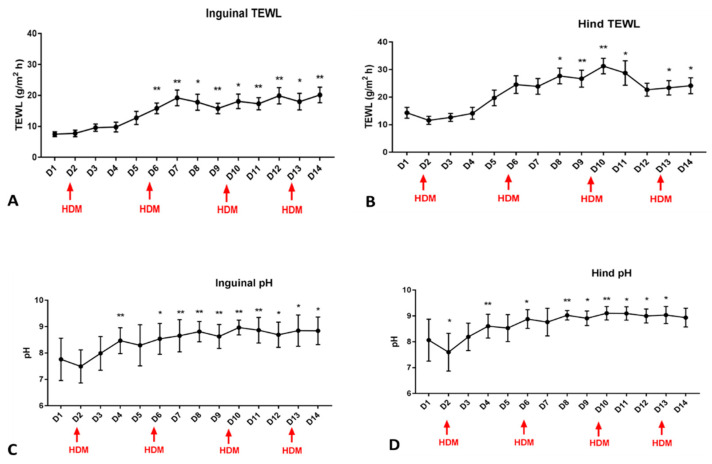
TEWL (**A**,**B**) and pH (**C**,**D**) during the course of the allergen challenge (red arrows show the days of allergen exposure). ANOVA showed a significant time effect for both the inguinal and hind leg (medial) for both pH and TEWL (*p* < 0.0001). Dunnett’s multiple comparisons test was performed to compare each day to baseline, and asterisks are used to mark the days when values were significantly higher compared to baseline. * is used for *p* values < 0.01 and ** is used for *p* values < 0.001.

The repeated measures ANOVA showed a significant effect of time for pH (*p* < 0.0001) and TEWL (*p* < 0.0001) with values being significantly increasing in both inguinal and medial thigh during the course of challenge (Figure 1). Dunnett’s multiple comparisons test was utilized to compare daily measurements to baseline and statistically significant increases were first noticed in pH (on day 4) and then in TEWL on day 6 (for the inguinal area) and day 8 (for TEWL on the medial thigh). From those time points, values remained significantly increased compared to the baseline for the remainder of the challenge.

### 3.2. Clinical Scores

**Figure 2:**
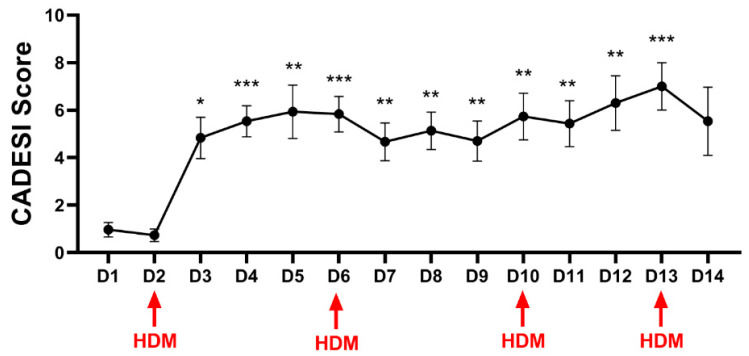
Means and standard deviations of the clinical scores (CADESI) of the inguinal area. Allergen challenges were carried out on days 2, 6, 10, and 13 as indicated by the red arrows. A statistically significant effect of time was reported (*p* < 0.0001). Dunnett’s multiple comparisons test was utilized to compare each day to the baseline, and a significant increase in clinical scores was seen starting on day 3. * is used for *p* values < 0.01, ** is used for *p* values < 0.001 and *** is used for *p* values < 0.0001.

A significant effect of time was also recorded for CADESI scores (*p* < 0.0001). Dunnett’s multiple comparisons test was utilized to compare each day to baseline, and a significant increase in clinical scores was seen starting on day 3, and scores remained significantly higher than at the baseline for the remainder of the study (Figure 2).

**Figure 3:**
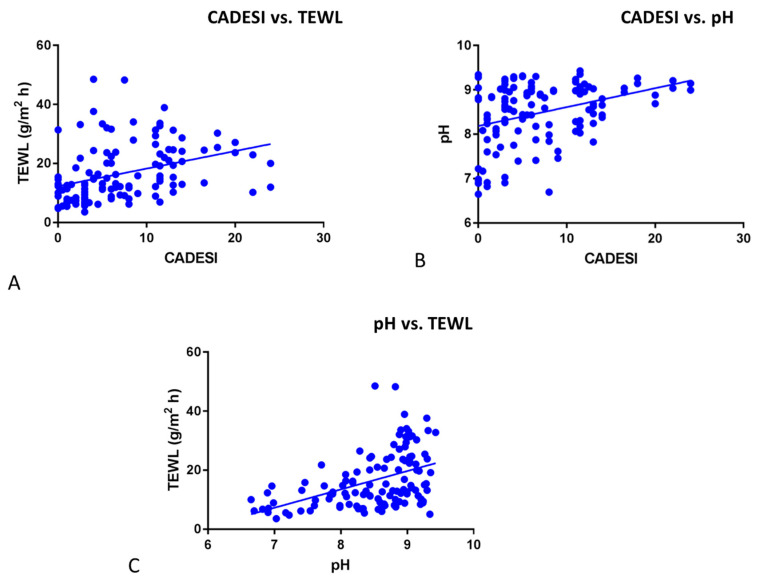
(**A**). Significant positive correlations were found between CADESI and TEWL (r = 0.36; *p* < 0.0001). (**B**). Significant positive correlations were found between CADESI and pH (r = 0.3556; *p* < 0.0001). (**C**). Significant positive correlations were found between pH and TEWL (r = 0.45; *p* < 0.0001).

Significant positive correlations were found between CADESI and TEWL (Figure 3A, r = 0.36; *p* < 0.0001), CADESI and pH (Figure 3B, r = 0.3556; *p* < 0.0001), and pH and TEWL Figure 3C, r = 0.45; *p* < 0.0001).

Vinegar water spray does not improve clinical signs or skin parameters during an allergen challenge, according to a randomized, double-blinded control study.

#### 3.2.1. Clinical Scores

**Figure 4:**
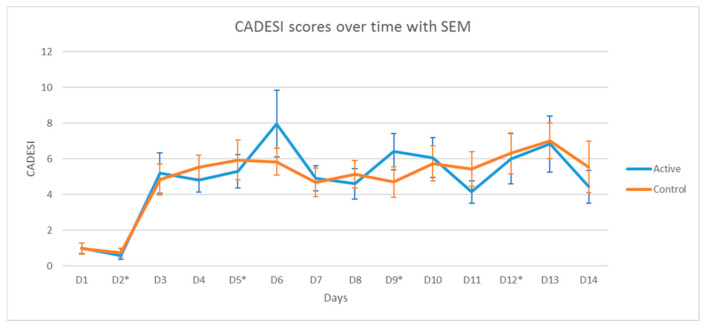
Means and standard errors of the mean for the clinical scores (CADESI) for the inguinal area. No significant differences were found between the site treated with the vinegar/water spray (active group) compared to the site treated with distilled water (control). Each dog received both treatments at different sites so that each dog functioned as its own control. * indicates dates of allergen challenge.

No significant differences in the severity of dermatitis were found between the sites treated with vinegar/water and the ones treated with water. Two-way repeated measure ANOVA only showed the significant effect of time (*p* < 0.0001) but no significant effect of group or group × time interactions. The scores of the inguinal area over the course of the allergen challenge are shown in Figure 4.

#### 3.2.2. pH

**Figure 5:**
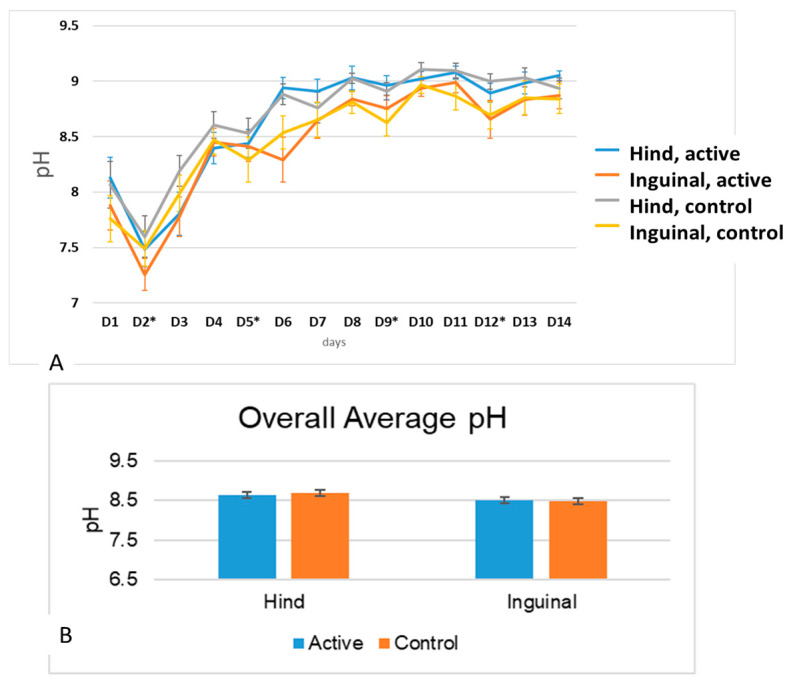
(**A**). Means and standard deviations of pH measurements during the course of allergen challenge for both the inguinal and medial thighs as the control and active sites. A significant effect of time was found (<0.0001), but no effects on group or group × time interaction was observed. The days of the allergen challenge are noted using an asterisk. (**B**) Means and standard errors of the means for all days combined pH measurements for hind leg and inguinal areas at the control and active sites. No significant differences were found between treatments on either site.

A significant increase in pH was found during the course of the challenge for both groups, both on the inguinal and medial thigh (Figure 5A). More specifically, for the hind leg (medial thigh), a two-way RM ANOVA showed no significant effect of group (*p* = 0.339), no significant effect of time (<0.0001), and no significant interaction group × time (*p* = 0.043), with the day 3 pH being higher in the control group than in the active group (*p* = 0.0027). For the inguinal area, a two-way RM ANOVA showed only a significant effect of time (*p* < 0.0001). A paired two-tailed *t*-test showed that the overall average pH was not significantly different between treatment sites (Figure 5B, hind leg, *p* = 0.3313; inguinal active vs. control: 0.7425).

#### 3.2.3. TEWL

**Figure 6:**
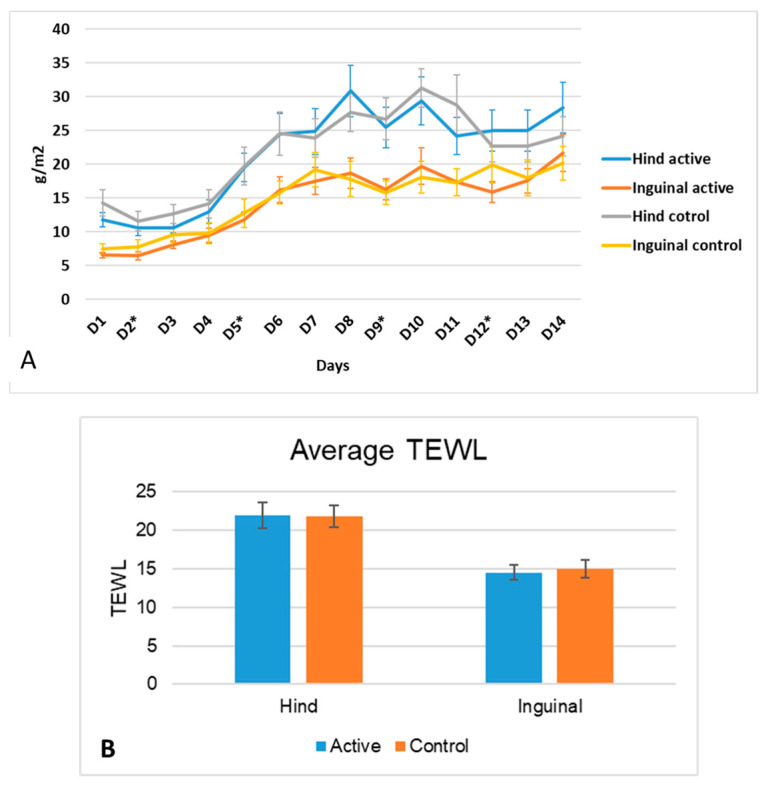
(**A**). Means and standard deviations of TEWL measurements during the course of allergen challenge for both inguinal and medial thigh of control and active sites. A significant effect of time was found (<0.0001), but no effects on group or group × time interaction were observed. The days of the allergen challenge are noted using an asterisk. (**B**) Means and the standard errors of the means for all the measurements of TEWL for the hind legs and inguinal areas at both the control and active sites. No significant differences were found between treatments for either site. Hind leg values were significantly higher than those of the inguinal area (*p* < 0.001).

For both the inguinal and hind legs, a two-way RM ANOVA only showed a significant increase in the significant effects of time (*p* < 0.0001), but the was no significance in group or group × time interactions (Figure 6A). Values for hind legs were overall significantly higher than the values for the inguinal areas (*p* < 0.001, Figure 6B). Paired two-tailed t-tests showed no significant differences between treatments for either site (hind leg *p* = 0.9; inguinal area *p* = 0.5) (Figure 6B).

**Figure 7:**
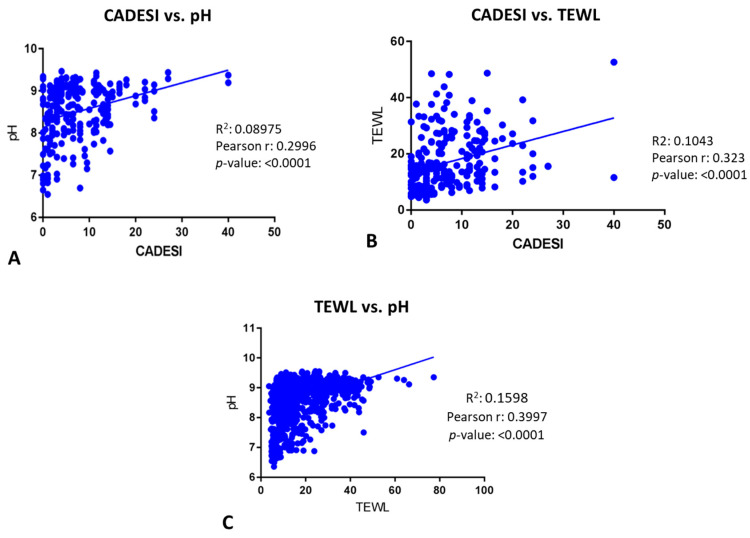
Correlations between CADESI, TEWL, and pH with all days compiled. (**A**) CADESI vs. pH were significantly correlated (*p* < 0.0001; r: 0.2996); (**B**) CADESI vs. TEWL were significantly correlated (*p* < 0.0001; r: 0.323); (**C**) TEWL vs. pH were significantly correlated (*p* < 0.0001; r: 0.3997).

When all times points were compiled, TEWL, pH, and CADESI were significantly positively correlated (Figure 7). More specifically, CADESI vs. pH (Figure 7A) had a *p* < 0.0001 and a Pearson’s r of 0.2996; CADESI vs. TEWL (Figure 7B) had a *p* < 0.0001 and a Pearson’s r of 0.323; and TEWL vs. pH (Figure 7B) had a *p* < 0.0001 and a Pearson’s r of 0.3997.

## 4. Discussion

Our study found that allergen exposure in atopic dogs leads to the increase in dermatitis scores, which positively correlate with increased TEWL and pH. It is interesting to point out the timings of these changes. Dermatitis scores were the first to increase upon allergen exposure and were significantly increased at day 3, followed by increased pH on day 4, and lastly, by increases in TEWL (on day 6 or day 7 depending on the site). Thus, it seems that, in terms of skin barrier parameters, pH is a more sensitive indicator of barrier disruption than TEWL.

pH is regulated by the many factors and bioproducts of filaggrin metabolism, and natural moisturizing factors are some of the most critical ones [30]. House dust mite challenges in dogs have been associated with a decrease in natural moisturizing factors due to allergic inflammation [31], and this could be one of the reasons why we observed the increased pH over the course of the allergen challenge. So, to explain the sequence of events over the course of the flare, we can say that pH is first parameter affected by allergic inflammation and that increased pH decreases lipid synthesis; this could be a mechanism for the subsequent increased TEWL. It is interesting to note an initial dip in pH on day 2. The exact reason for this is not known. One speculation is that the HDM solution was acidic, and this affected the pH when the dogs had not developed a full flare of atopic dermatitis. Later, as inflammation worsened, pH increased. As we did not measure the pH of the allergen solution, this is only speculation trying to explain why dogs in both groups experienced a decrease in pH during their first exposure to the allergen.

In this current study, we did not take samples to measure lipid changes as a result of the allergen challenge to be able to support this hypothesis, but it is known that increased TEWL correlates with decreased ceramides in atopic dogs [19]. As this colony of dogs has been retired, we are not able to further investigate lipid changes to support this hypothesis.

In our study, TEWL correlated with the severity of dermatitis and with pH, but it important to note that TEWL was reported to be one of the least reliable parameters of barrier functions in dogs [17] and in people due to the large variability and all the factors that can affect it [24,32].

pH instead appears to be a more reliable parameter in dogs [17]. Increased pH is also linked to disease severity in atopic people [12,33]. Other authors have reported an increase in pH with an increase in the severity of clinical scores in privately owned atopic dogs, but this correlation was applicable only for some body areas (e.g., lumbar areas and lateral thorax) and not others [18]. In such studies, there was no longitudinal assessment and no allergen challenge as the dogs were privately owned. In our study, we only measured pH in the inguinal areas and the medial thighs, as pH is also affected by other factors such as bathing routines [7] and diet [34]. In dogs, pH is also known to be affected by age, gender, body location, and coat color [35]. It is possible that all these features could have been a confounding aspect in the study on privately owned dogs as dogs of different breeds, ages and eating different diets were included. In our study, the dogs were all beagles and were kept in the same environment and were fed the same diet, possibly being one of the reasons for why we were able to show a consistent increase in pH. We focused on the areas where these dogs would develop clinical lesions of AD when exposed to the allergen, so we did not collect information on other body sites, which, in hindsight, would have been useful and interesting to have included.

The pH of normal canine skin has commonly been described as ranging from 5.5 to 8.3 [36,37]. In our allergic dogs, the pH was always alkaline and increased as the flare progressed. Age and regional differences have also been reported for cutaneous pH in dogs [38]. Thus, it is important to have baseline assessments and to compare changes to specific body sites and within the individual. Our dogs were all of the same age, and we specifically focused on two body areas throughout allergen exposure.

Acidification is considered an unexplored strategy to decrease the severity of atopic dermatitis and has been proposed as a strategy to halt the progression of disease [39]. We explored whether an old remedy like vinegar and water could help in terms of decreasing the severity of disease, but we did not find any benefits for either clinical signs or skin parameters. The likely reason for our results is that the ability of vinegar to lower the pH is short-lived and a more prolonged decrease in pH is likely needed to have a clinical benefit. In one study of normal dogs, undiluted vinegar maintained a pH of <5.0 for a mean of 3.8 h and <6.0 for a mean of 13 h [40]. In people, acidic baths (pH 3) are recommended for 40 min twice daily [41] in order to have any clinical benefit. This approach would not be sustainable for most dog owners.

In our study, we diluted vinegar with water to minimize irritation, but this increased the pH to 4. In hindsight, we could have used undiluted vinegar and used it more frequently, but we were hesitant to do this to minimize irritation as the dogs were simultaneously being challenged with house dust mites. Apple cider vinegar soaks (0.5%) have been recently reported as not effective as a sole treatment to improve skin barrier integrity in children [42]. The soaks were carried out for 10 min and, even if the vinegar was diluted, irritation was reported by the majority of patients.

The acidification of the stratum corneum is critical to improve lipid processing and decrease desquamation, thus improving skin barrier function [43]. Therefore, it is important to keep researching non-irritating, cost-effective, and sustainable strategies to lower pH in atopic dogs. As we face increased antibiotic resistance in Staphylococcal infections in dogs [44], it is important to pursue antibiotic-free ways to counteract Staphylococcus overgrowth. Increased pH leads to dysbiosis [45], and thus lowering pH is important for both skin barrier integrity and to minimize dysbiosis. It is hoped that this study can provide food for thought and inspire future longitudinal studies in clinically affected dogs comparing acidifying strategies with other treatments for AD and monitor how skin pH is impacted in the long term.

## 5. Conclusions

In conclusion, we found that allergen exposure in sensitized atopic dogs leads to the progressive increase in pH and TEWL, and these parameters positively correlate with the severity of dermatitis. The daily application of 50/50 vinegar water was not sufficient to lower the pH and had no positive effect on the dermatitis. Future studies should focus on alternative, sustainable strategies to lower pH in atopic dogs as a strategy to improve skin barrier function and prevent dysbiosis.

## Data Availability

Data will be made available upon reasonable request.
